# Draft genome sequences of *Alicyclobacillus* strains isolated from the Chinoike-Jigoku hot spring, Oita, Japan

**DOI:** 10.1128/mra.00118-26

**Published:** 2026-03-06

**Authors:** Yu Sato, Eriko Miyamoto, Yuna Sato, Shintaro Maeno

**Affiliations:** 1 Research Center for Thermotolerant Microbial Resources, Yamaguchi University 13150, Yamaguchi, Japan; 2 Department of Agriculture, Yamaguchi University 13150, Yamaguchi, Japan; 3 Graduate School of Science and Technology for Innovation, Yamaguchi University 13150, Yamaguchi, Japan; DOE Joint Genome Institute, Berkeley, California, USA

**Keywords:** draft genomes, *Alicyclobacillus*, isolate, Chinoike-Jigoku

## Abstract

The genus *Alicyclobacillus* is a thermoacidophilic bacterial genus found in diverse environments. Here, we report the draft genome sequence of *Alicyclobacillus* sp. strains CIJ1 and CIJ2 isolated from the Chinoike-Jigoku hot spring in Oita, Japan. Phylogenomic analyses suggest that these strains represent a candidate novel species within the genus.

## ANNOUNCEMENT

Members of the genus *Alicyclobacillus* are spore-forming Gram-positive rods that are typically acidophilic to moderately acidophilic and often exhibit thermophilic or thermotolerant growth (1). *Alicyclobacillus* species were historically classified within the genus *Bacillus* before being reclassified based on phylogenetic and chemotaxonomic features, including ω-alicyclic fatty acids (e.g., ω-cyclohexane fatty acids) in the cell membrane (2). *Alicyclobacillus* spp. have been isolated from diverse environments, such as soils (3–5), acidic fruit juices, and other beverages (6–12), human clinical specimens (13, 14), and hot springs (13, 15–17).

Several *Alicyclobacillus* species are important in food microbiology because their heat-resistant spores can survive pasteurization and spoil acidic beverages, sometimes producing off-flavor compounds, such as guaiacol (10, 18). On the other hand, other members of the genus are adapted to geothermal environments, such as hot springs. In these habitats, low pH and elevated temperatures provide ecological conditions consistent with their physiological traits. These environments likely represent natural reservoirs and sources of unexplored diversity within the genus.

Here, we report the draft genome sequences of two *Alicyclobacillus* strains, CIJ1 and CIJ2, isolated from a hot spring water sample collected at the Chinoike-Jigoku hot spring (Beppu, Oita Prefecture, Japan; 33.32°N, 131.47°E) in January 2023. One milliliter of hot spring water was spread on DSMZ medium 88 (pH 2.0) and incubated aerobically at 55°C for 1 week. Single colonies were purified by repeated streaking and cultured in DSMZ medium 88 at 55°C.

Genomic DNA was extracted using the Wizard Genomic DNA Purification Kit (Promega, Madison, WI, USA). Short-read sequencing libraries were prepared using the MGIEasy FS DNA Library Prep Set (MGI Tech Co., Shenzhen, China). The DNA libraries were sequenced on the DNBSEQ-G400RS platform (MGI Tech Co.) to generate paired-end sequences (150 bp × 2). A total of 4,795,509 and 3,214,148 paired-end reads were obtained for CIJ1 and CIJ2, respectively. Reads were trimmed and quality filtered using Platanus_trim v1.0.7 and assembled using Platanus_b v1.3.2 (19). Assemblies were annotated using the DDBJ Fast Annotation and Submission Tool (DFAST) v1.3.1 (20). Genome completeness and contamination were estimated using CheckM v1.2.2 (21) in DFAST. CheckM automatically selected a lineage-specific marker set corresponding to the *Alicyclobacillus acidocaldarius* clade (six reference genomes). Average nucleotide identity (ANI) values were calculated using pyANI version 0.3.0-alpha (22). The digital DNA-DNA hybridization (dDDH) values were estimated using the Genome-to-Genome Distance Calculator (GGDC) (23). Default parameters were used for all software unless otherwise specified.

**Fig 1:**
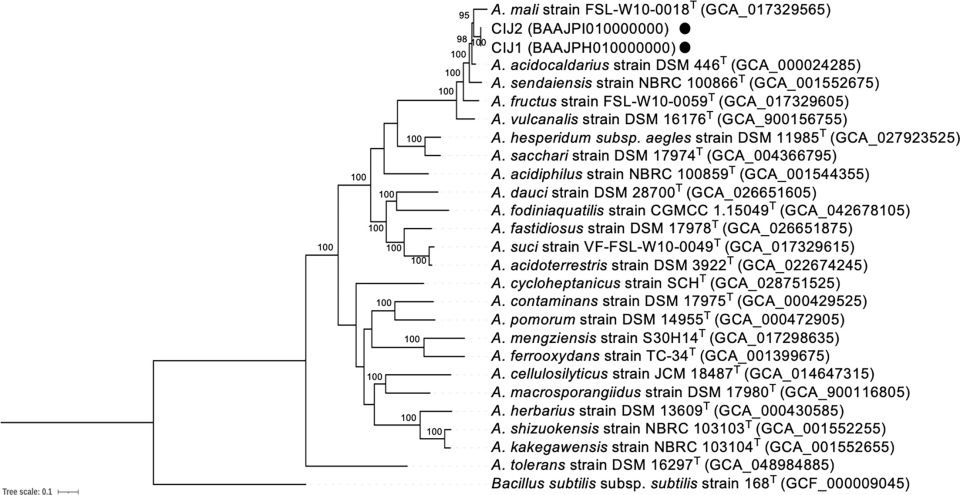
Maximum-likelihood tree based on 81 core genes among strains of *Alicyclobacillus* species. Labels for nodes with less than 80% bootstrap support were removed. The isolates in this study are highlighted with filled circles. The scale bar represents nucleotide substitutions per site. *Bacillus subtilis* subsp. *subtilis* strain 168^T^ was used as the outgroup. Scale bar, 0.1 substitutions per site.

The details of draft genomes for *Alicyclobacillus* sp. strains CIJ1 and CIJ2 are summarized in Table 1. Phylogenetic analysis was performed based on the concatenation of 81 core genes from each genome using UBCG2 (24). The two strains formed a distinct clade within the genus *Alicyclobacillus* (Fig. 1). The ANI and dDDH values between the genomes of strains CIJ1 and CIJ2 were 99.9% and 100%, respectively, indicating that CIJ1 and CIJ2 belong to the same species. The closest species were *A. mali* and *A. acidocaldarius*. ANI values with *A. mali* and *A. acidocaldarius* were 90.0% and 90.9%, respectively. In addition, the dDDH values with *A. mali* and *A. acidocaldarius* were 36.9% and 43.6%, respectively. These values are clearly below the accepted species delineation thresholds of 95%–96% ANI and 70% dDDH (23, 25), indicating that strains CIJ1 and CIJ2 represent a candidate for a novel species within the genus *Alicyclobacillus*.

**TABLE 1:** Information on genome sequences of strains in this study

Strain	CIJ1	CIJ2
Assembly length (bp)	3,015,628	3,017,064
No. of contigs	147	160
G + C (%)	61.8	61.8
Contigs N50 (bp)	187,884	172,877
CDS	2,973	2,984
rRNA	3	3
tRNA	64	64
CRISPR	4	4
Coding ratio (%)	87.6	87.7
CheckM completeness (%)	95.31	94.99
CheckM contamination (%)	4.46	4.46
SRA accession no.	DRX879802	DRX879803
GenBank accession no.	BAAJPH000000000.1	BAAJPI000000000.1

## Data Availability

The draft genome sequences of strains CIJ1 and CIJ2 are deposited at DDBJ/EMBL/GenBank under accession numbers BAAJPH010000000 and BAAJPI010000000, respectively. The raw sequencing reads are available in the Sequence Read Archive (SRA) under accession numbers DRX879802 and DRX879803, respectively.

## References

[1] Ciuffreda E, Bevilacqua A, Sinigaglia M, Corbo MR. 2015. Alicyclobacillus spp.: new insights on ecology and preserving food quality through new approaches. Microorganisms 3:625–640. doi:10.3390/microorganisms3040625 27682109 PMC5023266

[2] Wisotzkey JD, Jurtshuk P, Fox GE, Deinhard G, Poralla K. 1992. Comparative sequence analyses on the 16S rRNA (rDNA) of Bacillus acidocaldarius, Bacillus acidoterrestris, and Bacillus cycloheptanicus and proposal for creation of a new genus, Alicyclobacillus gen. nov. Int J Syst Bacteriol 42:263–269. doi:10.1099/00207713-42-2-263 1374624

[3] Groenewald WH, Gouws PA, Witthuhn RC. 2009. Isolation, identification and typification of Alicyclobacillus acidoterrestris and Alicyclobacillus acidocaldarius strains from orchard soil and the fruit processing environment in South Africa. Food Microbiol 26:71–76. doi:10.1016/j.fm.2008.07.008 19028308

[4] Goto K, Mochida K, Kato Y, Asahara M, Fujita R, An S-Y, Kasai H, Yokota A. 2007. Proposal of six species of moderately thermophilic, acidophilic, endospore-forming bacteria: Alicyclobacillus contaminans sp. nov., Alicyclobacillus fastidiosus sp. nov., Alicyclobacillus kakegawensis sp. nov., Alicyclobacillus macrosporangiidus sp. nov., Alicyclobacillus sacchari sp. nov. and Alicyclobacillus shizuokensis sp. nov. Int J Syst Evol Microbiol 57:1276–1285. doi:10.1099/ijs.0.64692-0 17551043

[5] Deinhard G, Blanz P, Poralla K, Altan E. 1987. Bacillus acidoterrestris sp. nov., a new thermotolerant acidophile isolated from different soils. Syst Appl Microbiol 10:47–53. doi:10.1016/S0723-2020(87)80009-7

[6] Walls I, Chuyate R. 2000. Isolation of Alicyclobacillus acidoterrestris from fruit juices. J AOAC Int 83:1115–1120. doi:10.1093/jaoac/83.5.1115 11048852

[7] Goto K, Matsubara H, Mochida K, Matsumura T, Hara Y, Niwa M, Yamasato K. 2002. Alicyclobacillus herbarius sp. nov., a novel bacterium containing omega-cycloheptane fatty acids, isolated from herbal tea. Int J Syst Evol Microbiol 52:109–113. doi:10.1099/00207713-52-1-109 11837292

[8] Roth K, Rana YS, Daeschel D, Kovac J, Worobo R, Snyder AB. 2021. Alicyclobacillus mali sp. nov., Alicyclobacillus suci sp. nov. and Alicyclobacillus fructus sp. nov., thermoacidophilic sporeforming bacteria isolated from fruit beverages. Int J Syst Evol Microbiol 71:005016. doi:10.1099/ijsem.0.005016 34550062

[9] Matsubara H, Goto K, Matsumura T, Mochida K, Iwaki M, Niwa M, Yamasato K. 2002. Alicyclobacillus acidiphilus sp. nov., a novel thermo-acidophilic, omega-alicyclic fatty acid-containing bacterium isolated from acidic beverages. Int J Syst Evol Microbiol 52:1681–1685. doi:10.1099/00207713-52-5-1681 12361274

[10] Luong TSV, Moir C, Bowman JP, Chandry PS. 2021. Heat resistance and genomics of spoilage Alicyclobacillus spp. Isolated from fruit juice and fruit-based beverages. Food Microbiol 94:103662. doi:10.1016/j.fm.2020.103662 33279087

[11] Oh S. 2017. Heat resistance of Alicyclobacillus isolated from fruit juices. Trends Agric Life Sci 55:23–27. doi:10.29335/tals.2017.55.23

[12] Groenewald WH, Gouws PA, Witthuhn RC. 2013. Thermal inactivation of Alicyclobacillus acidoterrestris spores isolated from a fruit processing plant and grape juice concentrate in South Africa. Afr J Microbiol Res 7:2736–2740. doi:10.5897/AJMR12.1789

[13] Kim MG, Lee J-C, Park D-J, Li W-J, Kim C-J. 2014. Alicyclobacillus tengchongensis sp. nov., a thermo-acidophilic bacterium isolated from hot spring soil. J Microbiol 52:884–889. doi:10.1007/s12275-014-3625-z 25037879

[14] Glaeser SP, Falsen E, Martin K, Kämpfer P. 2013. Alicyclobacillus consociatus sp. nov., isolated from a human clinical specimen. Int J Syst Evol Microbiol 63:3623–3627. doi:10.1099/ijs.0.048173-0 23606481

[15] Aulitto M, Gallo G, Puopolo R, Mormone A, Limauro D, Contursi P, Piochi M, Bartolucci S, Fiorentino G. 2021. Genomic insight of Alicyclobacillus mali FL18 isolated from an arsenic-rich hot spring. Front Microbiol 12:639697. doi:10.3389/fmicb.2021.639697 33897644 PMC8060452

[16] López G, Díaz-Cárdenas C, David Alzate J, Gonzalez LN, Shapiro N, Woyke T, Kyrpides NC, Restrepo S, Baena S. 2018. Description of Alicyclobacillus montanus sp. nov., a mixotrophic bacterium isolated from acidic hot springs. Int J Syst Evol Microbiol 68:1608–1615. doi:10.1099/ijsem.0.002718 29557767

[17] Simbahan J, Drijber R, Blum P. 2004. Alicyclobacillus vulcanalis sp. nov., a thermophilic, acidophilic bacterium isolated from Coso hot springs, California, USA. Int J Syst Evol Microbiol 54:1703–1707. doi:10.1099/ijs.0.03012-0 15388732

[18] Neggazi I, Colás-Medà P, Viñas I, Garza S, Alegre I. 2023. Occurrence of Alicyclobacillus acidoterrestris in pasteurized and high hydrostatic pressure-treated fruit juices and isolates’ characterization. Int J Food Microbiol 396:110197. doi:10.1016/j.ijfoodmicro.2023.110197 37084662

[19] Kajitani R, Yoshimura D, Ogura Y, Gotoh Y, Hayashi T, Itoh T. 2020. Platanus_B: an accurate de novo assembler for bacterial genomes using an iterative error-removal process. DNA Res 27:dsaa014. doi:10.1093/dnares/dsaa014 32658266 PMC7433917

[20] Tanizawa Y, Fujisawa T, Nakamura Y. 2018. DFAST: a flexible prokaryotic genome annotation pipeline for faster genome publication. Bioinformatics 34:1037–1039. doi:10.1093/bioinformatics/btx713 29106469 PMC5860143

[21] Parks DH, Imelfort M, Skennerton CT, Hugenholtz P, Tyson GW. 2015. CheckM: assessing the quality of microbial genomes recovered from isolates, single cells, and metagenomes. Genome Res 25:1043–1055. doi:10.1101/gr.186072.114 25977477 PMC4484387

[22] Pritchard L, Glover RH, Humphris S, Elphinstone JG, Toth IK. 2016. Genomics and taxonomy in diagnostics for food security: soft-rotting enterobacterial plant pathogens. Anal Methods 8:12–24. doi:10.1039/C5AY02550H

[23] Meier-Kolthoff JP, Carbasse JS, Peinado-Olarte RL, Göker M. 2022. TYGS and LPSN: a database tandem for fast and reliable genome-based classification and nomenclature of prokaryotes. Nucleic Acids Res 50:D801–D807. doi:10.1093/nar/gkab902 34634793 PMC8728197

[24] Kim J, Na S-I, Kim D, Chun J. 2021. UBCG2: up-to-date bacterial core genes and pipeline for phylogenomic analysis. J Microbiol 59:609–615. doi:10.1007/s12275-021-1231-4 34052993

[25] Meier-Kolthoff JP, Göker M, Spröer C, Klenk H-P. 2013. When should a DDH experiment be mandatory in microbial taxonomy? Arch Microbiol 195:413–418. doi:10.1007/s00203-013-0888-4 23591456

